# All ways lead to Rome: assembly of retromer on membranes with different sorting nexins

**DOI:** 10.1038/s41392-021-00561-z

**Published:** 2021-03-31

**Authors:** Xin Yong, Daniel D. Billadeau, Da Jia

**Affiliations:** 1 Key Laboratory of Birth Defects and Related Diseases of Women and Children, Department of Paediatrics, West China Second University Hospital, State Key Laboratory of Biotherapy and Collaborative Innovation Center of Biotherapy, Sichuan University, Chengdu, China; 2 Division of Oncology Research and Schulze Center for Novel Therapeutics, Mayo Clinic, Rochester, MN USA

**Keywords:** Cell biology, Structural biology

In a recent paper published in *Science Advances*, Leneva et al.^1^ present cryo-electron tomography structures of assembling metazoan and fungal core retromer complexes (VPS29/VPS35/VPS26) on membranes enriched in Øx(L/M/V) motif (where Ø is a bulky aromatic residue)-containing cargo and sorting nexin 3 (SNX3). The structures suggest that retromer forms a conserved arch-like scaffold that can incorporate different types of membrane adaptors and cargoes. More surprisingly, the SNX3-reotrmer complex is sufficient to induce membrane curvature and tubulation, in the absence of classical membrane curvature drivers, such as bin/amphiphysin/rvs-domain-containing sorting nexin protein (SNX-BARs).

The retromer complex is an evolutionarily-conserved assembly that plays a critical role in endosomal sorting—recycling endosomal proteins to other compartments including the *trans*-Golgi network and the plasma membrane. Retromer is thus critical for maintaining cellular homeostasis, and its mutations or dysfunctions have been observed in many pathophysiological conditions, such as Parkinson’s disease (PD), Alzheimer’s disease (AD), and amyotrophic lateral sclerosis (ALS). Interestingly, a recent genome-wide screen has identified retromer as one of the top candidates for SARS-CoV2 infection.^2^

Retromer often associates with members of the sorting nexin family to orchestrate the sorting of diverse cargo proteins into distinct trafficking routes. In fungi, retromer can associate with the SNX-BAR dimer, which, through its BAR domain, can generate/stabilize membrane curvature, or SNX3 (Grd19), which does not have the BAR domain. The situation is much more complicated in mammals as retromer can associate with SNX27, another SNX protein that lacks the BAR domain, in addition to the SNX-BAR dimer and SNX3.^3^ Furthermore, unlike their fungi orthologues, mammalian retromer does not tightly interact with SNX-BARs.

**Fig. 1:**
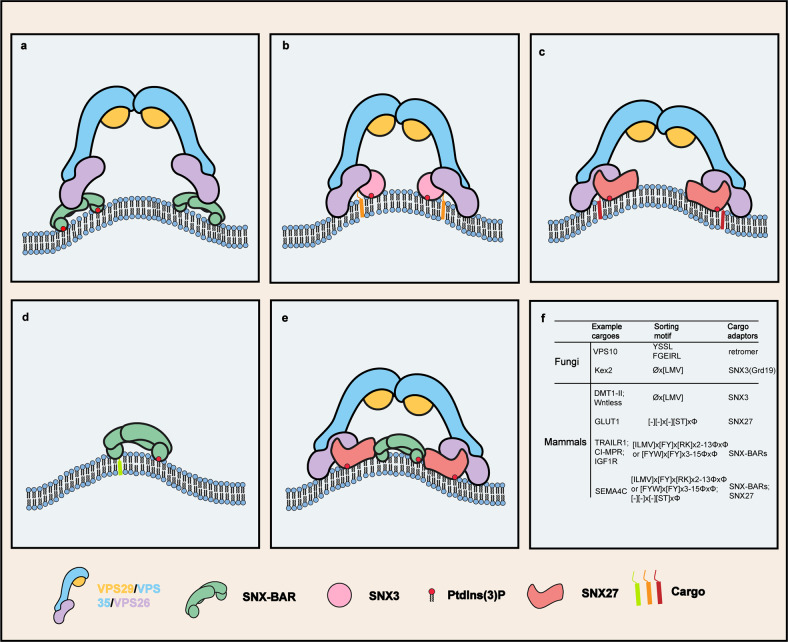
Models of retromer assembly on membranes. **a** A model of fungal SNX-BAR-retromer assembly on membrane. **b** A model of fungal and mammalian SNX3-retromer assembly on membrane. **c** A possible model of the SNX27-retromer assembly. **d** A possible model of SNX-BAR-mediated cargo recognition on membrane. **e** A possible model of SNX-BAR-SNX27-retromer super complex assembly. **f** Endosomal sorting motifs and their cargo adaptors in fungi and mammals. Φ: hydrophobic amino acids; −: negatively charged residues

Previous studies of the fungal SNX-BAR-retromer complex showed that SNX-BARs are the centerpieces to contact membrane, with both BAR and PX domains binding curved membranes (Fig. 1a).^4^ Retromer forms an arch-like structure on the layer of SNX-BARs. Two VPS35 molecules form a dimer and make up the legs of these arches. Such a conformation exposes VPS29 at the apex, making it accessible by diverse regulators. VPS26 is the only subunit that connects retromer with SNX-BARs. VPS26 forms homo-dimers to connect retromer arches with each other. The dimerization of VPS26 also bridges two neighboring SNX-BARs, thus stabilizing the curvatures. This structure provides significant insights into the mechanism where fungal retromer assembles on membranes with the assistance of SNX-BARs. Yet it fails to address how retromer promotes the transportation of cargoes together with non-BAR-containing adaptors.

On this point, Leneva et al. determined the architectures of metazoan and fungal SNX3-retromer assembly on membranes (Fig. 1b).^1^ Their cryo-electron tomography studies show that the complexes from both kingdoms adopt a similar structure.^1^ The SNX3-retromer is sufficient to induce membrane curvature, in contrary to the conventional wisdom that the BAR domain is required for membrane tabulation.^1^ Although analogous arches are observed, the assembly of SNX3-retromer on membranes distinguishes from that of SNX-BAR-retromer in multiple aspects.^1,4^ First, in the SNX3-retromer structure, retromer arches dock directly to the membranes via the VPS26 homo-dimers, with SNX3 playing a supplementary role. Second, whereas the cargo peptide is invisible in the SNX-BAR-retromer structure, the Øx(L/M/V) motifs bind to the interface between VPS26 and SNX3 in the SNX3-retromer structure. Last, SNX3 is found to contain a membrane insertion loop (MIL), which likely further enhances membrane curvature. The MIL motif can be found in many SNX proteins, including the SNX-BAR proteins SNX1 and SNX2, and non-BAR-containing proteins, such as SNX27.^1^

In addition to SNX3, SNX27 also participates in endocytic recycling together with retromer.^3^ Apart from the PX domain, SNX27 contains two additional domains including a postsynaptic density 95-discs large-zonula occludens (PDZ) and band4.1-ezrin-radixin-moesin (FERM) domain. Previous studies have established that SNX27 coordinates with retromer to transport cargoes encompassing PDZ-binding motifs (PDZbm) via its PDZ domain. As SNX27, similar to SNX3, does not contain a BAR domain, it remains to be determined whether the SNX27-retromer forms a structure analogous to that of the SNX3-retromer (Fig. 1c).

Mammalian retromer weakly interacts with SNX-BARs; however, both of them contact SNX27.^3^ As a result, we have recently proposed that SNX-BARs, SNX27, and retromer form a “supercomplex” to promote the endosome-to-plasma membrane recycling of specific cargoes.^5^ As both SNX-BARs (Fig. 1d) and retromer are able to contact membrane and induce curvature, it will be highly interesting to determine how the “supercomplex” assembles on the endosomal membrane and mediates cargo sorting (Fig. 1e, f).

Taken together, retromer is likely to adapt a similar arch-like architecture on membranes, and this arch structure and the ability to oligomerize renders retromer to sense and induce membrane curvature with BAR or non-BAR proteins. Investigation of retromer assembly not only allows us to gain molecular insights into diverse endocytic sorting routes, but also unveils potential targets for the treatment of multiple diseases. Further investigation is needed to understand how retromer cooperates with distinct adaptor proteins to mediate the sorting of different cargo proteins along diverse trafficking routes.
